# Upregulation of hypothalamic POMC neurons after biliary diversion in GK rats

**DOI:** 10.3389/fendo.2022.999928

**Published:** 2022-10-07

**Authors:** Shengnan Zhou, Weijie Chen, Xuesong Bai, Jiemin Chen, Qiang Xu, Liangbo Dong, Wei Chen, Qiang Qu, Xiaodong He

**Affiliations:** ^1^ Department of General Surgery, State Key Laboratory of Complex Severe and Rare Diseases, Peking Union Medical College Hospital, China Academy of Medical Science & Peking Union Medical College, Beijing, China; ^2^ Gastroenterology Department, State Key Laboratory of Complex Severe and Rare Diseases, Peking Union Medical College Hospital, China Academy of Medical Science & Peking Union Medical College, Beijing, China; ^3^ Clinical Nutrition Department, State Key Laboratory of Complex Severe and Rare Diseases, Peking Union Medical College Hospital, China Academy of Medical Science & Peking Union Medical College, Beijing, China

**Keywords:** biliary diversion, bile acids, FGF15, POMC neurons, metabolism

## Abstract

**Background:**

Bile acids are important signaling molecules that might activate hypothalamic neurons. This study aimed to investigate possible changes in hypothalamic pro-opiomelanocortin (POMC) neurons after biliary diversion in diabetic rats.

**Methods:**

Ten GK rats were randomly divided into the biliary diversion (BD) and sham groups. The glucose metabolism, hypothalamic POMC expression, serum bile acid profiles, and ileal bile acid-specific receptors of the two groups were analyzed.

**Results:**

Biliary diversion improved blood glucose (P = 0.001) and glucose tolerance (P = 0.001). RNA-Seq of the hypothalamus showed significantly upregulated expression of the POMC gene (log2-fold change = 4.1, P < 0.001), which also showed increased expression at the protein (P = 0.030) and mRNA (P = 0.004) levels. The POMC-derived neuropeptide α-melanocyte stimulating hormone (α-MSH) was also increased in the hypothalamus (2.21 ± 0.11 ng/g, P = 0.006). In addition, increased taurocholic acid (TCA) (108.05 ± 20.62 ng/mL, P = 0.003) and taurodeoxycholic acid (TDCA) (45.58 ± 2.74 ng/mL, P < 0.001) were found in the BD group and induced the enhanced secretion of fibroblast growth factor-15 (FGF15, 74.28 ± 3.44 pg/ml, P = 0.001) by activating farnesoid X receptor (FXR) that was over-expressed in the ileum.

**Conclusions:**

Hypothalamic POMC neurons were upregulated after BD, and the increased TCA, TDCA, and the downstream gut-derived hormone FGF15 might activate POMC neurons.

## Introduction

Type 2 diabetes mellitus (T2DM) is a major public health problem that causes vascular, renal, and neurologic complications (1). The number of T2DM patients is rising worldwide, although there are many treatment approaches available to control diabetes, such as sulphonylureas and metformin, exercise and diet. T2DM still causes high rates of disability and mortality year by year because of poor compliance of patients (2). Therefore, the search for new diabetes therapies is undoubtedly an active area of research and development.

Several randomized controlled trials and animal experiments have shown that bariatric surgery, including Roux-en-Y gastric bypass (RYGB) (3), sleeve gastrectomy (SG) (4) and biliopancreatic diversion (BPD) (5), is not only highly effective at producing weight loss but also results in significant improvement in T2DM (6). Moreover, many bariatric surgeries have been recommended as antidiabetes interventions for people with T2DM (7) and obesity (8), supported by the 2nd Diabetes Surgery Summit (DSS-II) guidelines (9).

The role bile acids (BAs) has been widely recognized recently (10). They are not only involved in the digestion of lipids but also act as signaling molecules that play an important role in glucose metabolism (11). It has been reported that BA levels are elevated following bariatric surgeries and could be potential mediators that contribute to metabolic homeostasis (12). In addition, biliary diversion surgery aimed at increasing enterohepatic circulation of BAs has been found to improve glucose levels (13), which supports the metabolic role of BAs. Farnesoid X receptor (FXR) and Takeda G-protein-coupled receptor 5 (TGR5) in the ileum are the BA-specific receptors that can be activated, leading to the secretion of gut-derived hormones, such as fibroblast growth factor-15 (FGF15) (14, 15) and glucagon-like peptide-1 (GLP-1) (16−18). BAs and downstream signaling molecules can activate the corresponding receptors that are present in the rat hypothalamus to regulate energy and glucose homeostasis (19, 20). Intracerebroventricular administration of FGF19 (ortholog of FGF15 in humans) has been found to increase energy expenditure and improve glucose tolerance through binding to the FGF receptor (21) stably mediated by Klotho (a transmembrane glycoprotein) (22).

The hypothalamus is critical for maintaining metabolic homeostasis. In particular, pro-opiomelanocortin (POMC) -expressing neurons (23) can produce a derived peptide, alpha-melanocyte-stimulating hormone (a-MSH) (24), leading to the activation of melanocortin 4 receptors (MC4Rs) (25) to exert a powerful effect on whole-body glucose regulation. According to Kalsbeek et al.'s reports, T2DM patients showed a decrease in the number of POMC-immunoreactive neurons (26). In addition, the prevalence of a specific heterozygous mutation in POMC was significantly higher in patients with early-onset obesity than in normal-weight controls (27), and heterozygous mutations in POMC may interfere with the effectiveness of bariatric surgeries (28). However, whether bariatric surgery leads to changes in these hypothalamic POMC neurons and the underlying mechanisms are unclear and imprecise.

Therefore, in an attempt to determine the changes in hypothalamic POMC neurons and the unique role of BA-related signaling pathways after bariatric surgery, we performed biliary diversion to the terminal ileum in Goto-Kakizaki (GK) rats.

## Materials and methods

### Animals

In this study, 10 GK rats (12-week-old male) weighing approximately 320 g were purchased from Junke Bioengineering Co., Ltd. (Nanjing, P.R. China). GK rats are a nonobese model of T2DM that present typical metabolic characteristics similar to those of human diabetes. The GK rats were housed in a climate-controlled environment with a 12-h light/dark cycle and were fed a standard chow diet and tap water in the laboratory animal center before being randomly allocated to the biliary diversion (BD) group (5 rats) or the sham group (5 rats). In addition, because rats are coprophagic animals and feces contain considerable amounts of bile acids, the rats were individually housed in metabolic cages during the study (**Supplementary Figure 1**). All experiments and surgical preparations were performed according to protocols approved by the Experimental Animal Welfare Ethics Committee.

### Operations

**Figure 1:**
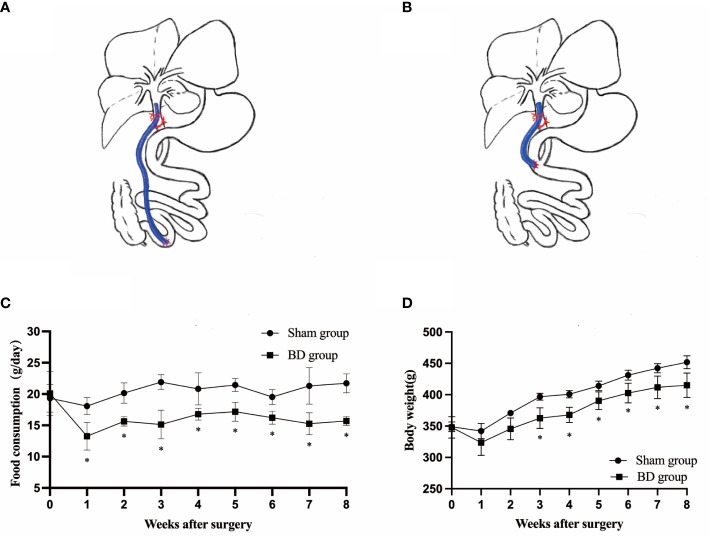
Surgical schematic and effects on body weight and food intake. **(A)** Sketch of biliary diversion of the common bile duct to the distal ileum. **(B)** Sketch of sham surgery with biliary diversion to the duodenum. **(C)** Average daily food intake in the two groups. **(D)** The body weight of the two groups. *P < 0.05.

The rats were fasted overnight (12 hours) and anesthetized by peritoneal injection of pentobarbital sodium that suited for experiments that focus on measurement of metabolic parameters (29) (the pentobarbital sodium concentration was 1%, and the injection dose was 35-40 mg per kilogram body weight). The biliary diversion operation is to transfer the bile in the common bile duct to the distal ileum (4 cm proximal to the ileocaecal valve) through a sterilized polytetrafluoroethylene (PTFE) tube with an inner diameter of 0.3 mm and outer diameter of 0.6 mm (**Figure 1A**). The abdominal wall was closed using simple, interrupted 3-0 polypropylene sutures. The sham operation involved the same procedures except that the distal end of the tube was fixed to the duodenum (parallel to the level of the ampulla of Vater) so that these rats also experienced surgical stress without physiologic biliary diversion (**Figure 1B**). All rats were fasted and deprived of water within 12 hours after surgery. The rats started to drink 12 hours after surgery and were fed enteral nutrient solution (10% ENSURE® Enteral Nutritional Powder (TP), ABBOTT LABORATORIES B.V.) from 24 hours after surgery to the third postoperative day. The surviving rats were maintained on a regular chow diet and water after surgery until the endpoint measurements.

### Food consumption and body weight

Rats were pair-fed to maintain the same food intake between the two groups and reduce the potential impact of food on glucose metabolism. The maximum food intake for 24 hours was measured without restriction from 8 am to 8 am the next day. Body weight was measured at 8 am after an overnight fast using an electronic scale.

### Glucose metabolism

Random blood glucose (RBG) levels in blood from the caudal vein were tested using a glucometer (Roche, Mannheim, Germany) at any time on any two days a week from 1 week before surgery to 8 weeks after surgery. Intraperitoneal glucose tolerance tests (IPGTT) were conducted before surgery and at 2, 4, 6, and 8 weeks after surgery. After fasting overnight (12 hours), the blood glucose levels of all rats were measured 0, 30, 60, 90 and 120 min using a glucometer after an intraperitoneal injection of dextrose (50%) at 2.0 mg/g body weight (30). The trapezoidal rule was used to determine the area under the curve (AUC) for the IPGTT (AUCIPGTT).

### RNA-sequencing of the hypothalamus

The rats were euthanized by anesthesia overdose at 8 postoperative weeks after an overnight fast to obtain the hypothalamus. Total ribonucleic acid was extracted from hypothalamus samples using TRIzol reagent (Invitrogen, CA, USA) according to the manufacturer's instructions. Transcriptional sequencing was implemented based on Illumina methods. An Agilent 2100 bioanalyzer was used to determine the quality and quantity of the extracted RNA from the samples. Briefly, cDNA libraries were prepared using a NEBNext® UltraTM Directional RNA Library ep Kit for Illumina®, and paired-end sequencing was performed on an Illumina HiSeq2000 sequencer. Clean read data for subsequent analysis were obtained after filtration of the raw data, a sequencing error rate check and a GC content distribution check. The clean sequence reads were aligned to the rat genome (Rnor 6.0.104) using HISAT2 2.1.0. Differentially expressed genes (DEGs) were identified using featureCounts (2.0.1) and the DESeq2 package (1.34.0) and judged by log2 (fold-change) ≥ 1 or log2 (fold-change) ≤ −1 and a P-adjusted value of < 0.05. Kyoto Encyclopedia of Genes and Genomes (KEGG) pathway analysis was performed using the R package (v4.1.0). RNA-Seq clean data were uploaded to the National Center for Biotechnology Information Gene Expression Omnibus database with the accession number PRJNA795651.

### Bile acid profiles

To identify the alteration in the bile acid profiles induced by biliary diversion, we measured and identified 18 bile acids in plasma samples collected from GK rats before surgery and 8 weeks postoperatively through targeted metabolomics analysis. At postoperative week 8, all rats were gavaged with 10% ENSURE® (1 ml/100 g body weight (31)) after fasting for 12 h. Blood was then collected from the angular vein at 2 hours after gavage. Targeted quantitative detection of serum bile acid was performed on an ultra-performance liquid chromatography-tandem mass spectrometry (UPLC−MS) platform (Waters Corporation, Milford, MA, USA) using the Waters ACQUITY UPLC I-class ULTRA high-performance liquid chromatography system combined with the Waters XEVO TQ-S tandem quadrupole mass spectrometry system. Data acquisition was carried out with MassLynx v 4.1 software (Waters Corporation, Milford, MA, USA).

### Biochemical tests

Serum insulin and glucagon levels were analyzed using enzyme-linked immunosorbent assay (ELISA) kits (Solarbio Life Science, Beijing, P. R. China). Serum FGF15 was evaluated using a Rat FGF15 ELISA Kit (Solarbio Life Science, Beijing, P. R. China), and serum GLP-1 was analyzed using a Rat GLP-1 ELISA Kit (CUSABIO, Wuhan, P. R. China). Serum and hypothalamus (1 mg of tissue in 9 ml of phosphate-buffered saline) α-MSH levels were assessed using a Rat α-MSH ELISA Kit (FineTest, Wuhan, China). All kits were used according to the manufacturers' guidelines.

### Western blotting

The hypothalamus and distal ileum were collected 8 weeks postsurgery and lysed in radioimmunoprecipitation assay buffer. Protein concentrations of the supernatant were quantified using the BCA Protein Assay Kit (Solarbio Life Science, Beijing, P. R. China), and equivalent amounts of protein were subjected to 10% sodium dodecyl sulfate−polyacrylamide gel electrophoresis and transferred to a 0.2 μm aperture polyvinylidene fluoride membrane. The membranes were blocked in 5% bovine serum albumin liquid and incubated with primary TGR5 (Abcam, Cambridge, UK), FXR (ABclonal, Wuhan, P.R. China), MC4R (ABclonal, Wuhan, P.R. China), Klotho (Proteintech, Wuhan, P.R. China), POMC (Wuhan, P.R. China) and β-actin (Cell Signaling Technology, Danvers, MA, USA) antibodies at 4 °C overnight. The membranes were washed three times with Tris-buffered saline and Tween 20, goat anti-rabbit (ABclonal, Wuhan, P.R. China) and goat anti-mouse (ABclonal, Wuhan, P.R. China) were incubated at room temperature for 1 hour with shaking. After three rinses with TBST solution, the membrane was scanned. The relative concentration of protein was quantified by densitometry using the Tanon Imaging System and ImageJ software.

### Real-time qPCR

Complementary DNA (cDNA) was synthesized in a TaqMan-based assay from 5 μg of extracted total RNA. The primer sequences are listed in **Table 1**. Real-time quantitative polymerase chain reaction (qPCR) analysis was performed using SYBR Green (Roche) on a LightCycler® 480 instrument (Roche, Germany). The mRNA expression of GAPDH was used for normalization, and 2^-△△Ct values were analyzed to determine the relative mRNA expression levels.

**Table 1:** Primer sequences Real-time qPCR.

Name	Sequences (5'→3')
TGR5 Forward	TACTCACAGGGTTGGCACTG
TGR5 Reverse	GTACCATTACAACGCGCTCAC
FXR Forward	GTACCATTACAACGCGCTCAC
FXR Reverse	AATTTCAGTTAACAAACATTCAGCC
MC4R Forward	TTGCTCGCATCCATTTGCAG
MC4R Reverse	TGCAAGCTGCCCAGATACAA
POMC Forward	TTCTGCTACAGTCGCTCAGG
POMC Reverse	GGATGCAAGCCAGCAGGT
Klotho Forward	TGGATCACCATTGACAACCC
Klotho Reverse	TTGGCGTGAGCCAAAAGTA
GAPDH Forward	GTCGGTGTGAACGGATTTGG
GAPDH Reverse	TCCCGTTGATGACCAGCTTC

### Immunohistochemistry

Hypothalamic tissue was fixed in 4% paraformaldehyde for 24 hours. Then, these tissues were embedded in paraffin and cut into 4-μm slices. For immunohistochemistry (IHC), the slides were incubated with antibodies against POMC, MC4R and Klotho and goat anti-rabbit and goat anti-mouse antibodies. Images were acquired and analyzed using a microscope and Case Viewer 2.4 software.

### Statistical analysis

The statistical analyses were performed using SPSS statistics software (version 25.0, IBM, Armonk, NY, USA) and Prism version 9 (GraphPad Prism, La Jolla, CA, USA). All quantitative data are expressed as the mean ± standard deviation. Student's t test (unpaired, two-tailed) and analysis of variance (ANOVA) test were used to compare the difference between the biliary diversion group and the sham group. The threshold for statistical significance was set at P < 0.05.

## Results

### Rat models

Biliary diversion and sham surgery were performed successfully in rat models. A BD rat died at postoperative week 2 because of intestinal obstruction due to abdominal adhesions, and a sham rat died at postoperative week 1 because of bile leakage. No other severe complications were observed.

### Food intake and body weight

Before surgery, the daily food intake (20.10 ± 3.49 g vs. 19.33 ± 2.20 g, P = 0.722) and body weight (347.75 ± 17.24 g vs. 348.88 ± 7.77 g, P = 0.911) were not significantly different between the BD group (4 rats) and sham group (4 rats). The consumption in the BD group remained significantly lower than that in the sham group at postoperative week 8 (15.60 ± 0.59 g vs. 21.73 ± 1.52 g, P = 0.007, **Figure 1C**). The body weight of the BD group and the sham group was not statistically significant until the third week after surgery (362.50 ± 16.52 g vs. 396.75 ± 5.50 g, P = 0.020) **Figure 1D** and the difference between the two groups on food intake (P = 0.013) and body weight (P < 0.001) was also statistically significant over time through ANOVA test.

### Effect on glucose metabolism

**Figure 2:**
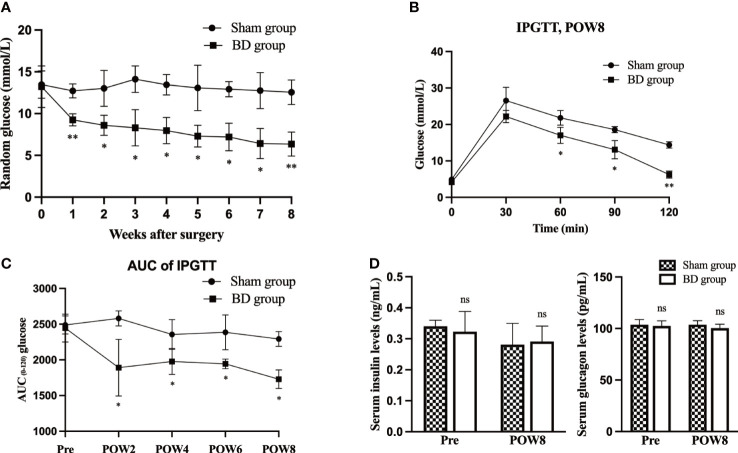
BD improved glucose homeostasis. **(A)** Random blood glucose (RBG) curves of the two groups presurgery and within 8 weeks after surgery. **(B)** Glycemic curves of intraperitoneal glucose tolerance tests (IPGTT) 8 weeks after surgery. **(C)** Area under the curve (AUC) measurements between 0 and 120 min in the IPGTT were calculated and compared between the two groups. **(D)** Serum insulin and glucagon levels in the two groups at presurgery and 8 weeks after surgery. *P < 0.05 and **P < 0.01. ns, not significant. POW, postoperative week. Pre, preoperation.

The RBG and the IPGTT were measured to evaluate the effects of biliary diversion on glucose homeostasis. The RBG levels in the BD group were significantly lower than those in the sham group (9.25 ± 0.72 mmol/L vs. 12.73 ± 0.84 mmol/L, P = 0.001) at postoperative week 8 (**Figure 2A**). The BD group also displayed improved glucose tolerance at 8 weeks postsurgery compared to the sham group (**Figure 2B**). In addition, the AUCIPGTT at 8 weeks (1729.00 ± 130.24 vs. 2292.00 ± 104.11, P = 0.001) postsurgery was significantly lower in the BD group than that in the sham group (**Figure 2C**), and the differences in RBG (P < 0.001) and AUCIPGTT (P < 0.001) between the two groups were also statistically significant over time. Moreover, the serum levels of insulin (0.29 ± 0.05 ng/ml vs. 0.28 ± 0.07 ng/ml, P = 0.823) and glucagon (100.26 ± 3.39 pg/ml vs. 103.63 ± 3.90 pg/ml, P = 0.269) did not differ significantly between the BD and sham groups (**Figure 2D**).

### Changes in hypothalamic POMC neurons

**Figure 3:**
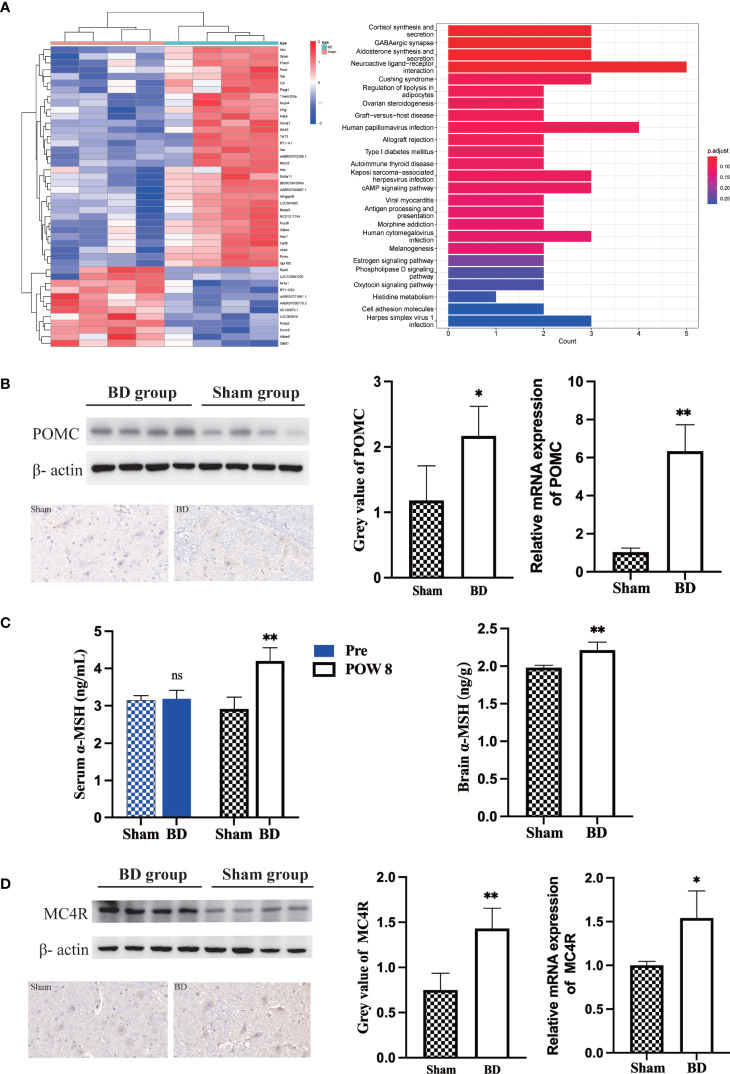
Biliary diversion altered hypothalamic gene expression and the related signaling pathway. **(A)** Left, clustered heatmap of the hypothalamic differentially expressed genes between the two groups. Right, the 25 most differentially expressed pathways between the two groups through KEGG analysis. **(B)** Western blots, IHC and RT−qPCR of POMC in the hypothalamus 8 weeks after surgery. **(C)** The concentration of α-MSH in the serum and hypothalamus 8 weeks after surgery in the two groups. **(D)** Western blots, IHC and RT−qPCR of MC4R in the hypothalamus 8 weeks after surgery. *P < 0.05. **P < 0.01. Scale bar = 10 μm. Sham, Sham group. BD, biliary diversion group.

Through RNA-seq of the hypothalamus (**Figure 3A**), we found that the POMC gene was significantly upregulated (log2-fold change = 4.1, P < 0.001) in the BD group compared to the sham group. According to KEGG pathway enrichment analysis (**Figure 3A**), the top four canonical pathways most increased in the BD group versus the sham group included cortisol synthesis and secretion, GABAergic synapse, aldosterone synthesis and secretion, and neuroactive ligand−receptor interaction signaling pathway.

The expression of POMC in the hypothalamus was measured by Western blotting. The relative expression level of hypothalamic POMC in the BD group (2.17 ± 0.45) was significantly higher than that in the sham group (1.18 ± 0.53, P = 0.030), and IHC also showed that the number of neuronal cells expressing POMC increased. For the BD group, RT−qPCR showed increased expression of the POMC gene in the hypothalamus (6.34 ± 1.29 vs. 1.03 ± 0.21, P = 0.004, **Figure 3B**). In addition, the concentrations of α-MSH, a POMC-derived neuropeptide, within the hypothalamus (2.21 ± 0.11 ng/g vs. 1.98 ± 0.03 ng/g, P = 0.006) and peripheral blood at 8 weeks postsurgery (4.20 ± 0.35 ng/mL vs. 2.91 ± 0.32 ng/mL, P = 0.002) were also significantly increased in the BD group (**Figure 3C**). The receptor of α-MSH in the hypothalamus-MC4R was also upregulated at the protein (1.43 ± 0.22 vs. 0.75 ± 0.19, P = 0.003) and mRNA levels (1.54 ± 0.31 vs. 1.00 ± 0.04, P = 0.038, **Figure 3D**).

### Bile acid alteration and its effects on the ileum

**Figure 4:**
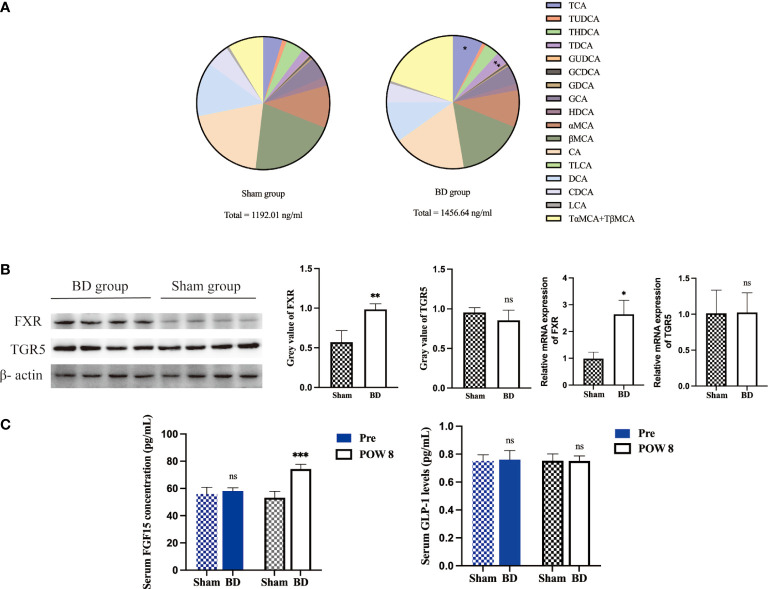
Altered bile acid pools and related signaling in the ileum after surgery. **(A)** Bile acid abundance and composition in the two groups 8 weeks after surgery. **(B)** Western blots and RT−qPCR of FXR and TGR5 in ileum tissue harvested from the two groups 8 weeks after surgery. **(C)** Serum FGF15 and GLP-1 concentrations presurgery and 8 weeks after surgery. *P < 0.05, **P < 0.01 and ***P < 0.001. ns, not significant. POW, postoperative week. Pre, preoperation. Sham, Sham group. BD, biliary diversion group.

The levels of serum total BA and 18 bile acid subclasses were not significantly different between the sham group and the BD group before surgery (**Table 2**). At 8 weeks after surgery, the levels of taurocholic acid (TCA, P = 0.003), taurodeoxycholic acid (TDCA, P < 0.001), and total bile acids (TBA, P = 0.035) were significantly higher in the BD group than in the sham group (**Figure 4A**).

**Table 2:** Serum bile acid species and levels (ng/ml) detected by targeted metabolomics.

Items	Before surgery			After surgery		
	Sham group (n=5)	BD group (n=5)	P value	Sham group (n=4)	BD group (n=4)	P value
TCA	53.15 ± 2.72	52.30 ± 3.51	0.679	55.71 ± 4.72	108.05 ± 20.62	0.003
TUDCA	15.34 ± 4.40	12.49 ± 2.13	0.228	13.31 ± 3.92	12.93 ± 2.52	0.874
THDCA	55.35 ± 5.76	56.32 ± 6.13	0.803	52.27 ± 2.68	51.49 ± 8.82	0.871
TDCA	21.81 ± 2.44	22.49 ± 2.23	0.656	25.20 ± 1.45	45.58 ± 2.74	0.000
GUDCA	1.62 ± 0.33	1.41 ± 0.26	0.275	1.29 ± 0.07	1.73 ± 0.93	0.379
GCDCA	5.95 ± 0.37	5.95 ± 0.37	0.990	6.55 ± 0.91	7.15 ± 1.11	0.435
GDCA	4.30 ± 1.18	5.28 ± 0.75	0.163	4.58 ± 0.41	4.66 ± 0.64	0.826
GCA	61.77 ± 13.94	60.37 ± 15.30	0.884	62.46 ± 16.87	67.00 ± 14.48	0.697
HDCA	22.32 ± 8.01	25.44 ± 6.23	0.511	25.06 ± 16.87	22.82 ± 11.83	0.769
αMCA	132.58 ± 38.37	123.59 ± 27.58	0.682	123.55 ± 29.77	131.71 ± 38.66	0.749
βMCA	234.86 ± 30.51	223.60 ± 37.89	0.619	248.21 ± 39.35	236.34 ± 40.87	0.691
CA	235.10 ± 40.85	238.38 ± 29.08	0.888	237.68 ± 12.06	259.32 ± 77.04	0.599
TLCA	0.28 ± 0.06	0.29 ± 0.07	0.902	0.20 ± 0.08	0.22 ± 0.06	0.722
DCA	148.20 ± 32.86	154.40 ± 32. 46	0.772	157.53 ± 20.81	143.13 ± 42.03	0.562
CDCA	68.71 ± 5.87	66.70 ± 6.22	0.613	67.33 ± 2.92	67.90 ± 6.69	0.882
LCA	8.34 ± 0.88	8.48 ± 0.96	0.811	7.73 ± 0.76	8.63 ± 1.62	0.238
TαMCA+TβMCA	118.13 ± 18.70	113.10 ± 18.03	0.676	103.35 ± 12.04	287.98 ± 176.69	0.128
TBA	1187.81 ± 86.57	1170.57 ± 47.33	0.706	1192.01 ± 35.17	1456.64 ± 150.22	0.035

TCA, taurocholic acid; TUDCA, tauroursodeoxycholic acid; THDCA, taurohyodeoxycholic acid.

TDCA, taurodeoxycholic acid; GUDCA, glycoursodeoxycholic acid; GCDCA, glycochenodeoxycholic acid.

GDCA, glycodeoxycholic acid; GCA, glycocholic acid; HDCA, hyodeoxycholic acid.

αMCA, alpha-muricholic acid; βMCA, beta-muricholic acid; CA, cholic acid.

TLCA, Taurolithocholic acid; DCA, deoxycholic acid.

CDCA, chenodeoxycholic acid; LCA, Lithocholic acid; TαMCA, tauro-alpha-muricholic acid.

TβMCA, tauro-beta-muricholic acid; TBA, total bile acids.

The expression of the bile acid-specific receptors FXR and TGR5 in the ileum was measured by Western blotting and RT−qPCR. The expression of FXR at the protein level (0.98 ± 0.07 vs. 0.57 ± 0.15, P = 0.002) and mRNA level (2.65 ± 0.52 vs. 0.98 ± 0.25, P = 0.004) in the BD group increased, whereas that of TGR5 showed no significant change at the protein level (0.85 ± 0.13 vs. 0.95 ± 0.06, P = 0.209) and mRNA level (1.02 ± 0.27 vs. 1.01 ± 1.32, P = 0.956, **Figure 4B**). In addition, the BD group displayed increased serum levels of FGF15 (74.28 ± 3.44 pg/ml vs. 53.19 ± 4.72 pg/ml, P = 0.001), which is a gut-derived hormone induced by FXR agonists, compared to the sham group. However, the serum GLP-1 concentration showed no significant differences between the two groups (0.75 ± 0.04 pg/ml vs. 0.75 ± 0.05 pg/ml, P = 0.981, **Figure 4C**).

### BAs-related receptors in the hypothalamus

**Figure 5:**
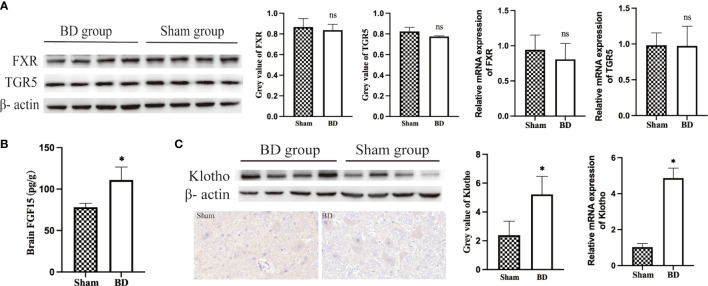
Bile acids and FGF15 receptors in the hypothalamus. **(A)** Western blots and RT−qPCR of bile acid receptors in the hypothalamus harvested from the two groups 8 weeks after surgery. **(B)** FGF15 concentration in brain tissue. **(C)** Western blots, IHC and RT−qPCR of Klotho in the hypothalamus harvested from the two groups 8 weeks after surgery. *P < 0.05. ns, not significant. Scale bar = 10 μm. Sham, Sham group. BD, biliary diversion group.

Given the increased level of bile acids and upregulation of POMC genes, bile acid signaling proteins in the hypothalamus were measured. The expression of BA-specific receptors FXR and TGR5 in the hypothalamus at the protein level (0.78 ± 0.01 vs. 0.82 ± 0.04, P = 0.570; 0.84 ± 0.06 vs. 0.87 ± 0.08, P = 0.059) and mRNA level (0.81 ± 0.23 vs. 0.94 ± 0.21, P = 0.413; 0.97 ± 0.23 vs. 0.98 ± 0.17, P = 0.949) was not significantly changed between the BD group and the sham group (**Figure 5A**). In addition, there was also no change in the expression of these two receptors at the gene level, which refuted the hypothesis that specific bile acids that crossed the blood−brain barrier act directly on the hypothalamus.

The BD group displayed significantly increased levels of FGF15 in serum (**Figure 4C**) and brain (110.91 ± 15.69 pg/g vs. 78.21 ± 4.58 pg/g, P = 0.007, **Figure 5B**), which could bind to the coreceptor Klotho in the hypothalamus. In view of the results of RNA-Seq that Klotho did not show a significant difference between the two groups, we detected the expression of Klotho through WB and RT−qPCR. Compared with the sham group, the BD group showed significantly increased expression of Klotho at the protein (5.22 ± 1.25 vs. 2.39 ± 0.97, P = 0.012) and mRNA levels (4.87 ± 0.56 vs. 1.03 ± 0.20, P < 0.001, **Figure 5C**). Moreover, IHC staining also showed that the expression of Klotho was increased in hypothalamic cells in the BD group compared with the sham group.

## Discussion

Biliary diversion resulted in improvements in glucose homeostasis and reduced food consumption and body weight of GK rats, which agreed with previous findings (32, 33). The levels of RBG and AUCIPGTT in the BD group decreased significantly after surgery and were lower than those in the sham group at postoperative week 8. The model procedure eliminated other factors that may affect metabolism, such as stomach capacity (34), alimentary path and pancreatic juice.

The hypothalamus is an appetite regulation center, and we found that anorexigenic (appetite-reducing) POMC neurons were upregulated at the gene, mRNA and protein levels through RNA-seq, RT−qPCR, WB and IHC. Over the years, the recognition of the importance of the hypothalamus in glucose metabolism and appetite regulation has inspired a new wave of research on neural pathways (35). POMC neurons, as one of the most intensively studied populations of hypothalamic neurons, have been found to express receptors corresponding to different circulatory factors, such as leptin (36), insulin (37), PYY (38) and GLP-1/2 (39). Some animal and clinical studies have focused on the abovementioned gut hormones and reported related alterations caused by bariatric surgery (40) (41). Our results showed that the serum levels of GLP-1, its upstream receptor TGR5, and its downstream molecule insulin were not significantly changed in the BD group, which may be because biliary diversion to the ileum did not change the rate of nutrient entry into the intestine (42), which suggests that there are other signaling molecules and pathways responsible for the upregulation of the POMC gene, such as the bile acids that have significantly changed after bariatric surgery.

Several studies have identified the metabolic benefit of increased bile acid through FXR and TGR5 in bariatric surgery. Through bile acid-targeted metabolomics, the serum concentrations of TCA and TDCA were found to be significantly increased in the BD group (43). TCA and TDCA were identified as FXR agonists (44), and the activation of FXR signaling potently induced gut-derived hormone secretion and improved glucose homeostasis in subjects who received RYGB (45) and SG (46). The expression of FXR in the distal ileum was significantly increased in the BD group. In addition, the serum concentrations of FGF15, which is secreted from the intestine into the blood induced by the activation of FXR, were also increased. This proved that elevated TCA and TDCA levels upregulated the intestinal FXR-FGF15 signaling pathway. However, among the two identified bile acid subtypes of FXR agonists, which subtype acts as the determinant in the regulation of glucose metabolism was not determined. Thus, the effect of altered bile acids observed in the BD group was predominantly on the bile acid-specific receptor FXR and the downstream of the intestinal endogenous molecule FGF15.

FGF15/19, as an intestinal hormone, has been shown to regulate glucose homeostasis (47) by binding to FGF receptors and the coreceptor Klotho, which are located in different tissues, such as the liver (48), adipose tissue (49), and the brain (50). Some studies have reported that FGF19 exerts insulin-like activity that increases protein and glycogen synthesis independent of insulin through the FGF19-βKlotho axis in the liver (51, 52). Recent studies focused on the mechanism of the metabolic benefits of FGF15 indicated that FGF15 suppressed glucagon secretion (53) and silenced hypothalamic AGRP/NPY neurons (54), and the beneficial metabolic effects of FGFs on body weight and insulin sensitivity were absent in mice lacking Klotho in the nervous system (55). Our results showed that the level of FGF15 was significantly increased and that the coreceptor Klotho was also highly expressed in the hypothalamus. Therefore, it is reasonable to assume that the increased FGF15 was associated with the upregulation of POMC neurons.

POMC-derived peptide, α-MSH, is a well-known regulator of the central nervous system that can bind to and activate MC4R neurons (56), which leads to appetite inhibition and body weight loss (56) and is responsible for improved glucose homeostasis (57). Our results suggest that bile diversion may related to the activated hypothalamic POMC-α-MSH-MC4R signaling pathway, which exerts appetite-reducing effects that have been well accepted. In addition, BD not only increased the concentration of α-MSH in hypothalamic tissues but also increased α-MSH levels in the peripheral circulatory system. Peripheral α-MSH increases glucose uptake in skeletal muscles and enhances glucose clearance by activating muscle MC5R and protein kinase A (58). Subsequently, Rodrigues AR et al. (59) found that α-MSH promoted white adipose tissue (WAT) browning and upregulated insulin-dependent glucose transporter type 4 (GLUT4) in WAT, which also increased glucose clearance.

There are a few limitations in our study. One major limitation is that our animal and molecular studies do not allow us to identify the causal relation of increased BAs, FGF15 and the upregulation of the POMC gene in the hypothalamus. More studies are needed to investigate the underlying neuronal circuitry engaged by FGF15, such as the antagonism of FGF receptors or BAs. In addition, the other limitations of animal experiments is species differences and the small sample size. Thus, predicting the potential pathways in humans based on the results of GK rats is complicated, and more research and additional data are needed to prove the underlying glucose homeostatic mechanism of bariatric surgery in the future on the basis of an enlarged sample size.

In conclusion, biliary diversion to the distal ileum led to decreased appetite and weight loss, as well as sustained hyperglycemia improvements in GK rats. POMC neurons are significantly overexpressed in the hypothalamus and might be activated by increased TCA, TDCA, and FGF15 levels after BD.

## Data availability statement

The original contributions presented in the study are included in the article/**Supplementary Material**, further inquiries can be directed to the corresponding author. The datasets of RNA-Seq in this study can be found in online repositories. The names of the repository/repositories and accession number(s) can be found below: https://dataview.ncbi.nlm.nih.gov/object/PRJNA795651.

## Ethics statement

The animal study was reviewed and approved by Experimental Animal Welfare Ethics Committee of Peking Union Medical College Hospital.

## Author contributions

SZ, WJC, and XH contributed to conception and design of the study. SZ, XB, QX, JC, and LD organized the database and performed the statistical analysis. SZ wrote the first draft of the manuscript. WC and QQ wrote sections of the manuscript. All authors contributed to the article and approved the submitted version.

## Funding

This work was supported by the Program Focus Health of Liver and Gallbladder in Elder (ZYJ201912), China Academy of Medical Science Innovation Fund for Medical Sciences (2021-1-I2M-022) and National Natural Science Foundation of China (81970763).

## Acknowledgments

We would like to thank Dr Kegong Tang and his colleagues, State Key Laboratory of Medical Molecular Biology, Institute of Basic Medical Sciences, Chinese Academy of Medical Sciences, Department of Biochemistry, Peking Union Medical College, for their invaluable technical efforts related to this project and the usage of the basic experimental facilities.

## Conflict of interest

The authors declare that the research was conducted in the absence of any commercial or financial relationships that could be construed as a potential conflict of interest.

## Publisher’s note

All claims expressed in this article are solely those of the authors and do not necessarily represent those of their affiliated organizations, or those of the publisher, the editors and the reviewers. Any product that may be evaluated in this article, or claim that may be made by its manufacturer, is not guaranteed or endorsed by the publisher.

## Supplementary material

Supplementary Figure 1 Metabolic cages used for housing GK rats.
