# Supplementary figures and images for: Upregulation of hypothalamic POMC neurons after biliary diversion in GK rats
Source: Front Endocrinol (Lausanne). 2022 Oct 7;13:999928. doi: 10.3389/fendo.2022.999928 (PMC9585246; doi:10.3389/fendo.2022.999928)

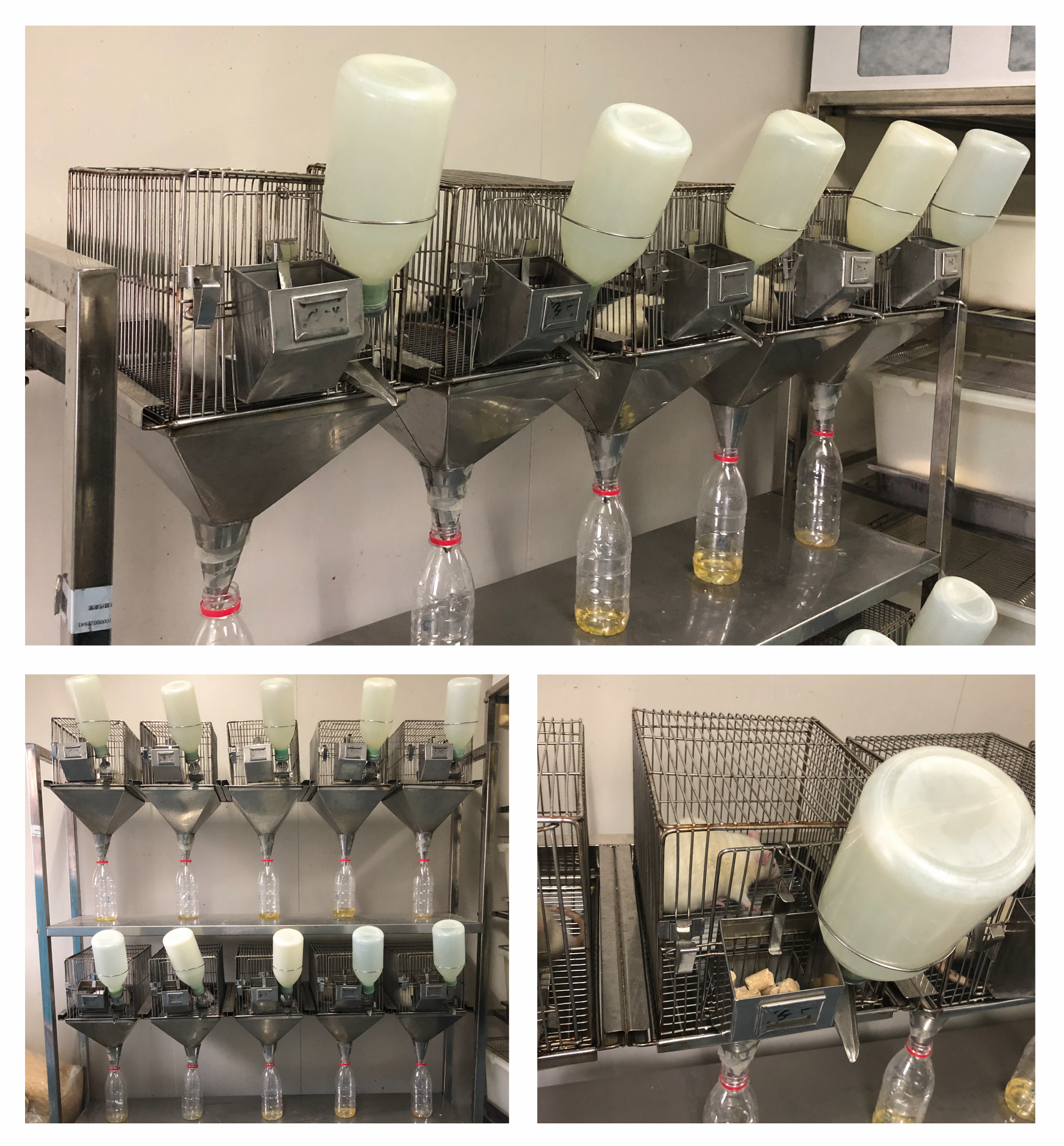

Supplement: Supplementary Figure 1 — Metabolic cages used for housing GK rats. [file Image_1.png]
